# Assessing the implementation processes of a large-scale, multi-year quality improvement initiative: survey of health care providers

**DOI:** 10.1186/s12913-018-3045-6

**Published:** 2018-04-03

**Authors:** Donna Goodridge, Masud Rana, Elizabeth L. Harrison, Thomas Rotter, Roy Dobson, Gary Groot, Sonia Udod, Joshua Lloyd

**Affiliations:** 1 College of Medicine, University of Saskatchewan, Saskatoon, Canada; 2 Department of Community Health and Epidemiology, College of Medicine, University of Saskatchewan, Saskatoon, Canada; 3 School of Physical Therapy, College of Medicine, University of Saskatchewan, Saskatoon, Canada; 4 Health Quality Programs, Queen’s University, Kingston, Canada; 5 College of Pharmacy and Nutrition, University of Saskatchewan, Saskatoon, Canada; 6 Faculty of Nursing, University of Manitoba, Winnipeg, Canada

**Keywords:** Quality improvement, Large-scale transformation, Lean, Health care professionals, Nurses, Normalization process theory, Survey

## Abstract

**Background:**

Beginning in 2012, Lean was introduced to improve health care quality and promote patient-centredness throughout the province of Saskatchewan, Canada with the aim of producing coordinated, system-wide change. Significant investments have been made in training and implementation, although limited evaluation of the outcomes have been reported. In order to better understand the complex influences that make innovations such as Lean “workable” in practice, Normalization Process Theory guided this study. The objectives of the study were to: a) evaluate the implementation processes associated with Lean implementation in the Saskatchewan health care system from the perspectives of health care professionals; and b) identify demographic, training and role variables associated with normalization of Lean.

**Methods:**

Licensed health care professionals were invited through their professional associations to complete a cross-sectional, modified, online version of the NoMAD questionnaire in March, 2016. Analysis was based on 1032 completed surveys. Descriptive and univariate analyses were conducted. Multivariate multinomial regressions were used to quantify the associations between five NoMAD items representing the four Normalization Process Theory constructs (coherence, cognitive participation, collective action and reflexive monitoring).

**Results:**

More than 75% of respondents indicated that neither sufficient training nor resources (collective action) had been made available to them for the implementation of Lean. Compared to other providers, nurses were more likely to report that Lean increased their workload. Significant differences in responses were evident between: leaders vs. direct care providers; nurses vs. other health professionals; and providers who reported increased workload as a result of Lean vs. those who did not. There were no associations between responses to normalization construct proxy items and: completion of introductory Lean training; participation in Lean activities; age group; years of professional experience; or employment status (full-time or part-time). Lean leader training was positively associated with proxy items reflecting coherence, cognitive participation and reflexive monitoring.

**Conclusions:**

From the perspectives of the cross-section of health care professionals responding to this survey, major gaps remain in embedding Lean into healthcare. Strategies that address the challenges faced by nurses and direct care providers, in particular, are needed if intended goals are to be achieved.

**Electronic supplementary material:**

The online version of this article (10.1186/s12913-018-3045-6) contains supplementary material, which is available to authorized users.

## Background

The province-wide introduction of Lean as a strategy to improve health care quality and promote patient-centredness was initially launched by the Government of Saskatchewan of Canada in 2012 [1]. Lean refers to a set of operating philosophies, tools and activities that help to create maximum value for patients by reducing sources of waste in a process [2, 3].This large scale transformation was aimed at effecting coordinated, system-wide change affecting multiple organizations and care providers [4]. In keeping with recommendations that active implementation strategies are essential to producing a coherent and multilevel approach to health care transformation [5–8], significant investments in implementation were directed towards training initiatives, particularly at the senior leadership level. Initial implementation of Lean was delivered through a paid consultant and later transitioned to local leadership. More than 1000 projects [1] have been undertaken throughout the publicly-funded health regions in this province of 1.1 million people and the government re-confirmed its commitment to support the ongoing use of Lean in its 2016 strategic plan [1].

Because Lean is a “high-touch, high-maintenance enterprise” [9, 10], the costs of implementation are high. Estimates of the investment by the provincial government in Lean since 2010 are in excess of $44 million dollars [11], contributing to the politicization of this quality improvement strategy [12] in a setting where public tax revenues fund the health care system. The Provincial Auditor has noted the absence of evaluation on the outcomes of Lean implementation [13], although the government reported that the initial outcomes of Lean implementation have been promising. Evidence supporting the use of Lean in health care, however, is weak, with a recent systematic review [14] concluding that Lean interventions: were not associated with patient satisfaction or health outcomes; were negatively associated with financial costs and worker satisfaction; and had potential but inconsistent benefits for safety and patient flow.

Achieving the stated goals of Lean implementation to improve health care quality and promote patient-centredness is contingent upon health care professionals working individually and collectively to embed this new strategy into their organizational and professional contexts [15]. Given the lack of consensus about how to measure the success of complex interventions in general [15, 16], Normalization Process Theory (NPT) [17] offers an approach to understanding the complex influences that make innovations such as the introduction of Lean “workable” in practice settings [18, 19]. The overall aim of this study was to examine the extent to which Lean has become embedded within health care after four years of implementation using an online survey of licensed health care providers. The objectives of the study were to: a) evaluate the implementation processes associated with Lean implementation in the Saskatchewan health care system from the perspectives of health care professionals; and b) identify demographic, training and role variables associated with normalization of Lean.

Although there is a substantial literature relating to implementation of complex interventions in service organizations, understanding the ways in which large-scale transformations are embedded and integrated into evolving and diverse practice settings remains a challenge.^8^ Normalization Process Theory (NPT) [17] is one approach to better understanding the work required at individual and collective levels to implement complex interventions. NPT addresses the generative processes that underpin: a) *implementation* (bringing practices into action); b) *embedding* (when practices are routinely embedded into the everyday work of individuals and groups); and c) *integration* (when practices are reproduced and sustained within the social matrices of an organization). Four core constructs have been identified that represent the different kinds of work people and organizations do to implement a new practice such as Lean; each construct is comprised of four distinct components (Additional file 1: Table S1).

According to the NPT model, the organizing structures and social norms of practice environments influence the way in which new practices are accommodated and directly affect the organizing factors of a practice, while the group process and conventions affect the way in which a practice is produced and reproduced in actual patterns of interpersonal behavior [17]. Organizing structure and social norms, together with group processes and conventions, affect perceptions of coherence (the meaningful qualities of a practices) of an intervention to those involved in implementation. *Coherence* affects and is affected *by cognitive participation* (the enrolment and engagement of individuals and groups), which has a reciprocal relationship with the practices already existing (*collective action*). *Reflexive monitoring* refers to the ways a practice is understood and assessed by those involved. This understanding and assessment serves to modify both organizing structures and social norms as well as group processes and conventions.

A substantial body of evidence supports NPT as a means of explaining processes associated with implementation of complex interventions ranging from telecare and e-health [20, 21] to maternity services [22] to management of depression [23, 24]. Recently, the NoMAD instrument was developed to operationalize and measure the core constructs of NPT with the intent that this tool could be customized for a wide range of purposes and across different settings [15].

## Methods

### Ethics and consent

A cross-sectional, online survey design was used. The questionnaire was administered during the month of March, 2016 for a period of 30 days. Ethical approval (BEH 13–294) was obtained from the University of Saskatchewan. Consent to participate was implied by completion of the survey.

### Measures

The NoMAD tool is a 23 item instrument using a 5-point Likert scale developed to assess healthcare professionals’ perspectives on the processes related to implement complex interventions in practice [15]. Surveys are amongst the methods that can be appropriately employed to study the process of improvement initiatives [25]. Given that the development and testing of tools designed to evaluate the processes involved in quality improvement initiatives such as Lean remains an emerging focus of research [25], the decision to use the NoMAD was made on the basis of this conceptual fit of this tool with the overall objectives of this study. Descriptive analysis and psychometric testing of the NoMAD was recently undertaken by the scale authors using 831 completed questionnaires, with confirmatory factor analysis demonstrating the model achieved an acceptable fit (personal communication, T. Finch, September 2017). Construct validity of the four NPT constructs was supported with internal consistencies ranging from 0.65 (reflexive monitoring) to 0.81 (cognitive participation.

The NoMAD Survey core constructs and components are detailed in Additional file 1: Table S1. Adaptations to the survey were made by the team to reflect the local context and nature of intervention. Because the original item “I will continue to support [the intervention]” implied that providers, in fact, were supportive, and this had not been established, the following revision was used: “I support the use of Lean in health care”. Another adaptation was made to the original item “I have confidence in other people’s ability to use [the intervention]”. Given that we were uncertain about the extent to which Lean practices were actually being implemented in practice by the range of practitioners we planned to survey, this item was modified to read: “I am confident in the skills of people leading the use of Lean”. Finally, the original item worded “I am aware of reports about the effects of [the intervention]” was revised to ask about the outcomes, rather than the effects, of Lean. The remaining 20 items were used directly from the original tool.

At the beginning of the questionnaire, definitions of Lean principles and activities were included to orient and ensure respondents were able to distinguish the intervention from others that may have been occurring in their workplaces. To ensure the definitions reflected the nature of Lean as implemented within the province, the following definitions were identified in consultation with the provincial Health Quality Council (personal communication, T. Verrall, February 2016), the agency responsible for coordinating Lean implementation in the province during the period 2013–15. *Lean principles* were defined as: a dedication to continuous improvement; focus on eliminating waste; improving the flow of patient; providers and supplies; and ensuring all processes add value to the customer. Problems are identified and addressed by front line members as Lean suggests that the people doing the work are best suited to solving the problem. An extensive list of *Lean activities* included activities such as Daily Visual Management, Wall Walks, and Rapid Process Improvement Workshops (RPIW).

Respondents were initially asked to identify their level of familiarity with these Lean principles and activities on a 1–10 Likert-type scale and to identify the types of Lean training received. Respondents identified the types of Lean activities they had participated in as health care providers, the duration to which they had been exposed to Lean and the extent to which implementation of Lean had affected their perceived workload. Key demographic and professional characteristics were included based upon our previous work with Lean and health care professionals [26, 27].

### Population and sample

Health care providers registered with professional licensing bodies throughout the province were invited to participate in the online survey. Application to conduct the survey with members was approved by the following licensing bodies: the Saskatchewan Registered Nurses Association (*n* = 10,000 with 4800 opting into receiving any surveys); the Saskatchewan Association of Licensed Practical Nurses (*n* = 3400); the Registered Psychiatric Nurses of Saskatchewan (*n* = 879); the Saskatchewan College of Physiotherapists (*n* = 600); the Saskatchewan College of Pharmacy Professionals (*n* = 264 hospital-based pharmacists); the Saskatchewan Medical Association (*n* = 2144); and the Saskatchewan Dietitians Association (*n* = 355). Several other professional associations with fewer members did not have the resources to participate in the survey. Given that it was not possible to ascertain a priori which professionals may have been using Lean in their practices and thus target the sample only to those providers involved with Lean, the decision was made to survey all members, with the exception of pharmacists who could be identified as practicing in a hospital setting and thus were exposed to Lean implementation. The medical association provided a link to the survey in their bi-weekly newsletters, while the remaining licensing bodies sent a personal email to members containing a link to the survey.

### Process

In collaboration with the Social Science Research Laboratory at the University of Saskatchewan, the online questionnaire was pilot-tested with 10 professionals representing the disciplines included in the survey. Minor editorial revisions were made to the questionnaire, which was hosted by Qualtrics™ and was available for 30 days. Reminder emails and announcements were sent 2 weeks following the initial invitations.

### Statistical analysis

Statistical analyses were conducted using SAS/STAT® software, Version 9.4 of the SAS System for Windows. Frequencies were computed for all variables and presented as observed/total and percentage. Responses to the main survey items were collapsed for clarity of interpretation into three categories: Agree (strongly agree or agree), Neutral (neither agree nor disagree), and Disagree (strongly disagree or disagree). We summarized responses of Nurses (who comprise the largest occupational category of health care providers) and compared them to the responses of other Health Professionals using chi-square tests to examine differences between the two groups.

In order to identify demographic, training and role variables associated with normalization of Lean, five items from the survey were selected by the research team on the basis of conceptual importance as outcome variables to represent each of the NPT constructs [14]. These items were: “I can see the potential value of Lean for my work” (Coherence item reflecting internalization); “I support the use of Lean in health care” (Cognitive Participation item reflecting activation); “Sufficient training is provided to enable health care providers to implement Lean in health care” (Collective Action item reflecting skillset workability); “Sufficient resources are available to support the implementation of Lean in health care” (Collective Action item reflecting contextual integration); and “The people I work with believe that Lean is worthwhile” (Reflexive Monitoring item reflecting communal appraisal).

Univariate analyses were conducted to determine simple associations. Multinomial logistic regression was used to investigate the relationships between the multivariable relationships between the five variables selected as measures of Lean normalization and variables representing respondents’ professional and personal characteristics, their Lean-related training, and the extent of experience with Lean events. Descriptive analyses were performed to ensure all assumptions underlying the use of multinomial regression were met. The strength of associations was measured based on the Odds Ratio (O-R). The sensitivity of estimated O-R was assessed using bootstrap analysis and 95% percentile confidence intervals (CI) of the estimates were computed from 5000 bootstrap sample. We also reported model-based adjusted *p*-value for each estimate; adjustment was made following Benjamini and Hochberg [28].

## Results

**Fig. 1:**
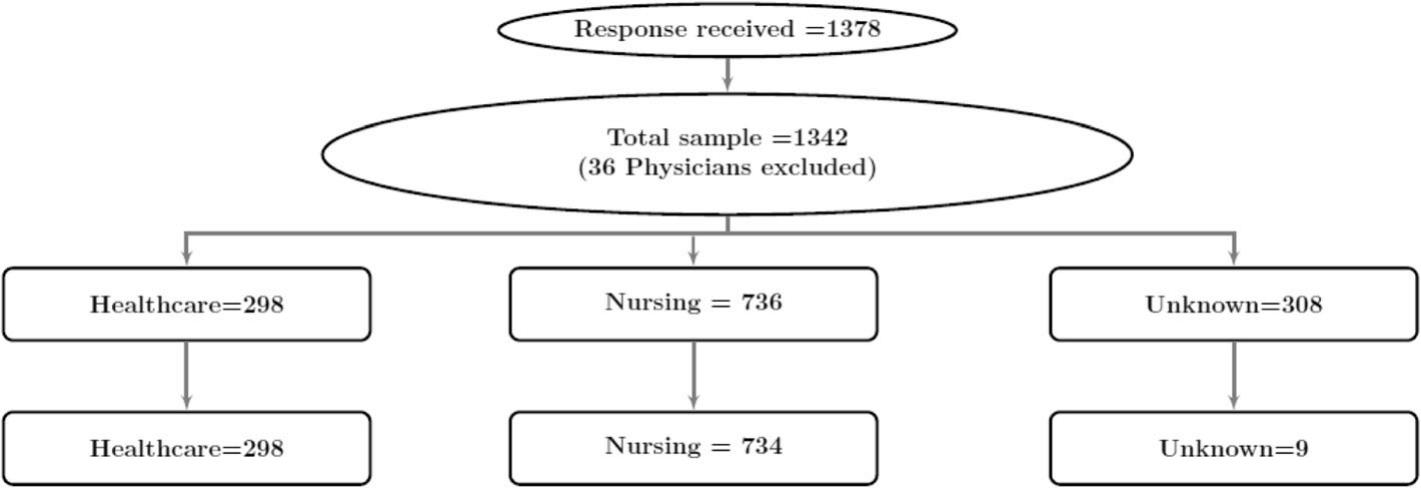
Flow Diagram of Survey Responses

A total of 1378 health care providers completed the survey. Response rate by professional group is reported in Additional file 2: Table S2. Because there were only 35 physician responses, the decision was made to exclude their responses from the analysis. Participants’ profession was not disclosed in 308 surveys, of which 299 had at least 25% missing values to the NoMAD items, leaving nine surveys to enter the study. These nine subjects, along with three more with similar level of missing responses, were excluded from the analysis. On the basis of similarities in role, the various classifications of nurses were combined into one category (Nursing), while the remaining providers were aggregated into a second category labelled health professionals (HP). Responses were retained for: 734 nurses (395 Registered Nurses; 314 Licensed Practical Nurses; 25 Registered Psychiatric Nurses) and 298 Health Professionals (44 Registered Dietitians; 67 Physiotherapists; 56 Occupational Therapists; 131 Pharmacists). Details of the sample selection process has been summarized in a flow-diagram in Fig. 1.

Reflecting the composition of the healthcare workforce in this province [29], most respondents were female and direct care providers. Nurse respondents were older, less likely to work full-time and more likely to work in acute care settings than HPs. There were no significant differences between nurses and HPs in terms of years of experience, primary role or geographic location, with respondents from settings outside of the large urban centers being well-represented (44.1%). A full description of respondent characteristics is located in Table 1.

**Table 1:** Characteristics of Respondents

	Nursing (*n* = 734)	Health Professionals (*n* = 298)	*P*-value
Sex			0.0001
Female	683 (94.7)	242 (82.3)	
Male	38 (5.3)	52 (17.7)	
Age Category			0.0001
< 30 years	98 (14.1)	61 (21.4)	
31–45 years	277 (39.7)	136 (47.7)	
> 45 years	322 (46.2)	88 (30.9)	
Employment Status			0.0001
Full-time	456 (62.1)	235 (78.9)	
Part-time	207 (28.2)	55 (18.5)	
Casual	71 (9.7)	8 (2.7)	
Years of Experience			0.37
0–5 years	139 (19.0)	71 (23.8)	
6–10 years	137 (18.7)	54 (18.1)	
11–15 years	109 (14.9)	40 (13.4)	
> 15 years	348 (47.5)	133 (44.6)	
Primary Role			0.03
Direct Care	603 (82.2)	222 (75.0)	
Leadership	76 (10.4)	46 (15.5)	
Other	55 (7.5)	28 (9.5)	
Practice Setting			0.0001
Acute care	392 (53.9)	126 (42.9)	
Long-term care	123 (16.9)	22 (7.5)	
Other	213 (29.3)	146 (49.7)	
Health Region			0.34
Urban	408 (55.9)	174 (59.2)	
Small Urban/Rural/Remote	322 (44.1)	120 (40.8)	

In terms of Lean familiarity, integration and the impact on workload (Table 2), wide variability was reported within both groups. While approximately two-thirds of respondents reported no, little or moderate familiarity with Lean principles and activities, 32.6% self-rated their familiarity as substantial or complete. Lean was not currently a part of their work for more than one-third of respondents, and 32% of nurses and 26% of HPS believed that Lean was likely to become part of their normal work in the future. Significantly different (*p* < 0.0001) impacts on workload were reported by nurses and HPs, with 75% of nurses and 42% of HPs reporting that Lean had increased their workload.

**Table 2:** Lean Familiarity, Integration and Impact on Workload

	Nursing (*n* = 734)	Health Professionals (*n* = 298)	*P*-value
Familiarity with Lean principles and/or activities			0.0001
None or little (1–4)	217 (29.6)	58 (19.5)	
Somewhat (5–7)	260 (35.4)	150 (50.3)	
Substantial or complete (8–11)	257 (35.0)	90 (30.2)	
Currently normal part of work			0.18
Not at all or very little (1–3)	251 (34.2)	112 (37.6)	
Somewhat (4–5)	235 (32.0)	78 (26.2)	
Completely (6–11)	248 (33.8)	108 (36.2)	
Will become normal part of work			0.005
Unlikely (1–2)	227 (31.0)	70 (23.7)	
Somewhat likely (3–5)	275 (37.6)	103 (34.8)	
Very likely (6–11)	230 (31.4)	123 (41.6)	
Impact of Lean on Workload			0.0001
No impact	123 (16.8)	141 (47.6)	
Increased somewhat	328 (44.8)	82 (27.7)	
Increased substantially	221 (30.2)	42 (14.2)	
Decreased somewhat or substantially	61 (8.3)	31 (10.5)	

Participation in Lean training and activities is reported in Table 3. No significant differences in Lean training were noted between nurses and HPs. While the majority of respondents had attended an introductory session (Kaizen Basics), 17.5% reported they had received no training at all in Lean principles or activities. A separate cross-tabulation (not shown) to examine possible collinearity between reporting a primary work role as a senior or front-line leader and completion of Lean leadership training indicated that close to 70% of leaders had *not* received this specialized training. The majority of respondents (91%) had participated in at least one Lean activity. The Lean activity most frequently reported by respondents was the Visibility Wall/Wall Walk (71–73%).

**Table 3:** Participation in Lean Training and Activities

	Nursing (*n* = 734)	Health Professionals (*n* = 298)	*P*-value
Lean Training (%)			
Kaizen Basics	556 (75.9)	232 (77.9)	0.49
Lean Leadership Certification	37 (5.1)	21 (7.1)	0.21
Other Lean training	74 (10.1)	40 (13.4)	0.12
No Training	124 (16.9)	57 (19.1)	0.40
Participation in Lean Events			
Visibility Walls/Wall walks	538 (73.4)	212 (71.1)	0.46
Daily Visual Management (Huddles)	415 (56.6)	175 (58.7)	0.54
5S	347 (47.3)	132 (44.3)	0.37
Kanban	265 (36.2)	65 (21.8)	0.0001
RPIW	189 (25.8)	97 (32.6)	0.03
Mistake Proofing	116 (15.8)	46 (15.4)	0.88
3P	98 (13.4)	40 (13.4)	0.98
Other	148 (20.2)	77 (25.8)	0.05
No Participation	84 (11.4)	58 (19.5)	0.001
First Exposure to Lean Training, activities or principles			0.05
< 1 year ago	26 (3.6)	21 (7.2)	
About 1 year ago	68 (9.3)	22 (7.5)	
About 2 years ago	200 (27.3)	70 (23.9)	
3 or more years ago	439 (59.9)	180 (61.4)	
Impact of Lean on Workload			0.0001
No impact	123 (16.8)	141 (47.6)	
Increased somewhat	328 (44.8)	82 (27.7)	
Increased substantially	221 (30.2)	42 (14.2)	
Decreased somewhat or substantially	61 (8.3)	31 (10.5)	

Additional file 3 Table S3 displays responses to the NoMAD construct items. Because further psychometric testing is still required on subscale and cumulative scores, items are reported individually under the constructs and not tallied. Significant differences (*p* < 0.05 or greater) in responses were noted between nurses and HPs on 13 of the 20 items, with nurses displaying less propensity towards normalization in all cases.

Results for the multivariate multinomial regression models for each of the five selected outcome variables are reported in Tables 4, 5, 6, 7 and 8. Professional characteristics were significantly associated with agreement with the NPT items used as measures of Lean normalization. Having a leadership role, compared to a direct care role, was one of the strongest predictor variables for the cognitive participation, coherence, and reflexive monitoring models. Respondents in leadership roles were significantly more likely to agree than direct care providers with the following survey items: I support the use of Lean in health care (O.R. = 10.29, 95% C.I. 5.65–24.7), I can see the potential value of Lean for my work (O.*R* = 7.83, 95% C.I. 4.29–17.8) and that the people they worked with believed Lean was worthwhile (O.R. = 4.33, 95% C.I. 2.24–9.74). There were no statistically significant differences between those in a leadership role and those in direct care in terms of responses to the sufficient training or sufficient resources (collective action) items.

**Table 4:** Multivariate Multinomial Model: Adjusted Associations between Coherence Item and Demographic, Personal and Practice Variables

	I can see the potential value of Lean for my work					
	Neutral			Agree		
	Odds Ratio	95% CI	P-value	Odds Ratio	95% CI	*P*-value
Profession						
Nursing	1.0	–	–	1.0	–	–
Health Care	2.15	(1.38, 3.49)	0.004	2.57	(1.73, 4.12)	0.001
Age group						
≤ 30 years	1.0	–	–	1.0	–	–
31–45 years	0.76	(0.35 1.55)	0.68	1.23	(0.61, 2.51)	0.77
> 45 years	0.91	(0.38, 2.08)	0.95	1.17	(0.49, 2.75)	0.88
Employment Status						
Full-Time	1.0	–	–	1.0	–	–
Part-Time/Casual	0.81	(0.53, 1.23)	0.49	0.87	(0.57, 1.30)	0.74
Years of experience						
0–5 years	1.0	–	–	1.0	–	–
6–10 years	1.00	(0.48, 2.02)	0.92	0.67	(0.32, 1.33)	0.44
11–15 years	1.12	(0.51, 2.61)	0.99	0.65	(0.31, 1.38)	0.26
15+ years	1.05	(0.49, 2.40)	0.99	0.56	(0.25, 1.18)	0.44
Primary Role						
Direct Care	1.0	–	–	1.0	–	–
Leadership	2.58	(1.14, 6.17)	0.06	7.83	(4.29, 17.8)	0.001
Other	2.39	(1.20, 5.12)	0.05	1.77	(0.91, 3.65)	0.26
Practice Setting						
Acute	1.0	–	–	1.0	–	–
Long-term Care	1.44	(0.81, 2.53)	0.41	1.31	(0.75, 2.27)	0.54
Other	1.30	(0.84, 2.06)	0.44	1.01	(0.65, 1.56)	0.99
Location						
Urban	1.0	–	–	1.0	–	–
Rural/Remote	1.23	(0.84, 1.84)	0.47	1.71	(1.19, 2.57)	0.03
Familiarity with Lean						
Complete	1.0	–	–	1.0	–	–
Somewhat	1.85	(1.21, 3.03)	0.03	0.77	(0.50, 1.15)	0.41
Training						
None	4.34	(1.68, 30.7)	0.05	0.49	(0.17, 2.00)	0.23
Kaizen Basics	3.10	(1.24, 19.1)	0.15	0.81	(0.35, 1.84)	0.77
Leadership	3.11	(0.64, 17.8)	0.26	3.72	(1.53, 18.7)	0.08
Participation						
None (vs. any)	1.32	(0.63, 2.56)	0.54	0.98	(0.44, 2.20)	0.21
Visibility wall	1.14	(0.66, 2.10)	0.99	1.46	(0.89, 2.63)	0.88
5S	1.02	(0.66, 1.54)	0.88	1.08	(0.71, 1.66)	0.32
RPIW	1.21	(0.76, 2.06)	0.77	1.41	(0.96, 2.42)	0.99
Impact of Lean on Workload						
No impact/Decreased	1.0	–	–	1.0	–	–
Increased somewhat	0.62	(0.38, 0.97)	0.12	0.33	(0.21, 0.49)	0.001
Increased substantially	0.39	(0.22, 0.63)	0.002	0.08	(0.04, 0.13)	0.001
First Exposure to Lean						
About 2 years ago	1.0	–		1.0	–	–
3 or more years ago	0.93	(0.62, 1.40)	0.88	0.97	(0.65, 1.45)	0.99

**Table 5:** Multivariate Multinomial Model: Adjusted Associations between Cognitive Participation Item and Demographic, Personal and Practice Variables

	I support the use of Lean in health care					
	Neutral			Agree		
	Odds Ratio	95% CI	*P*-value	Odds Ratio	95% CI	*P*-value
Profession						
Nursing	1.0	–	–	1.0	–	–
Health Care	2.53	(1.69, 3.98)	0.0001	4.11	(2.70, 7.20)	0.0001
Age group						
≤ 30 years	1.0	–	–	1.0	–	–
31–45 years	1.55	(0.84, 2.99)	0.37	1.82	(0.85, 3.98)	0.37
> 45 years	1.99	(0.95, 4.49)	0.21	2.15	(0.87, 5.71)	0.32
Employment Status						
Full-Time	1.0	–	–	1.0	–	–
Part-Time/Casual	1.07	(0.72, 1.57)	0.84	0.96	(0.59, 1.55)	0.94
Years of experience						
0–5 years	1.0	–	–	1.0	–	–
6–10 years	0.71	(0.37, 1.30)	0.58	0.77	(0.35, 1.63)	0.72
11–15 years	0.73	(0.36, 1.51)	0.21	0.79	(0.33, 1.82)	0.54
15+ years	0.52	(0.24, 1.04)	0.49	0.66	(0.28, 1.54)	0.69
Primary Role						
Direct Care	1.0	–	–	1.0	–	–
Leadership	2.05	(0.90, 4.67)	0.21	10.29	(5.65, 24.7)	0.0001
Other	1.30	(0.61, 2.66)	0.60	1.46	(0.71, 3.06)	0.53
Practice Setting						
Acute	1.0	–	–	1.0	–	–
Long-term Care	1.17	(0.66, 2.03)	0.72	2.44	(1.36, 4.68)	0.02
Other	1.19	(0.77, 1.82)	0.60	1.09	(0.65, 1.82)	0.84
Location						
Urban	1.0	–	–	1.0	–	–
Rural/Remote	1.29	(0.90, 1.88)	0.37	1.89	(1.25, 2.99)	0.02
Familiarity with Lean						
Complete	1.0	–	–	1.0	–	–
Somewhat	1.20	(0.80, 1.85)	0.59	0.59	(0.36, 0.93)	0.08
Training						
None	0.92	(0.39, 2.54)	0.96	0.28	(0.09, 0.68)	0.05
Kaizen Basics	0.73	(0.31, 1.85)	0.60	0.54	(0.22, 1.21)	0.37
Leadership	0.92	(1.47, 4.15)	0.94	3.45	(1.24, 14.9)	0.08
Participation						
None (vs. any)	1.63	(0.83, 3.46)	0.94	0.72	(0.29, 1.70)	0.49
Visibility wall	1.55	(0.98, 3.09)	0.96	1.31	(0.75, 2.57)	0.98
5S	1.05	(0.68, 1.54)	0.21	1.00	(0.61, 1.63)	0.54
RPIW	1.03	(0.66, 1.65)	0.37	1.33	(0.77, 2.26)	0.60
Impact of Lean on Workload						
No impact/Decreased	1.0	–	–	1.0	–	–
Increased somewhat	0.61	(0.39, 0.96)	0.60	0.19	(0.11, 0.29)	0.08
Increased substantially	0.26	(0.14, 0.41)	0.0001	0.05	(0.02, 0.09)	0.0001
First Exposure to Lean						
About 2 years ago	1.0			1.0		
3 or more years ago	0.79	(0.53, 1.15)	0.40	0.76	(0.48, 1.17)	0.40

**Table 6:** Multivariate Multinomial Model: Adjusted Associations between Collective Action (Training) Item and Demographic, Personal and Practice Variables

	Sufficient training is provided					
	Neutral			Agree		
	Odds Ratio	95% CI	P-value	Odds Ratio	95% CI	P-value
Profession						
Nursing	1.0	–	–	1.0	–	–
Health Care	2.00	(1.37, 303)	0.01	1.57	(1.02, 2.45)	0.23
Age group						
≤ 30 years	1.0	–	–	1.0	–	–
31–45 years	1.23	(0.66, 2.29)	0.75	0.99	(0.47, 2.11)	0.99
> 45 years	1.14	(0.54, 2.37)	0.85	0.66	(0.26, 1.63)	0.64
Employment Status						
Full-Time	1.0	–	–	1.0	–	–
Part-Time/Casual	0.85	(0.59, 1.21)	0.68	1.27	(0.85, 1.92)	0.53
Years of experience						
0–5 years	1.0	–	–	1.0	–	–
6–10 years	0.65	(0.34, 1.18)	0.99	1.13	(0.54, 2.44)	0.92
11–15 years	0.98	(0.48, 1.94)	0.75	1.08	(0.47, 2.53)	0.42
15+ years	1.26	(0.65, 2.51)	0.45	1.81	(0.82, 4.38)	0.85
Primary Role						
Direct Care	1.0	–	–	1.0	–	–
Leadership	0.57	(0.27, 1.08)	0.32	1.21	(0.64, 2.29)	0.75
Other	0.73	(0.37, 1.32)	0.64	0.99	(0.47, 1.98)	0.99
Practice Setting						
Acute	1.0	–	–	1.0	–	–
Long-term Care	1.00	(0.59, 1.65)	0.99	0.90	(0.49, 1.52)	0.85
Other	1.06	(0.72, 1.56)	0.88	0.96	(0.62, 1.47)	0.92
Location						
Urban	1.0	–	–	1.0	–	–
Rural/Remote	1.27	(0.91, 1.82)	0.45	1.52	(1.06, 2.23)	0.19
Familiarity with Lean						
Complete	1.0	–	–	1.0	–	–
Somewhat	0.88	(0.59, 1.28)	0.75	0.39	(0.26, 0.56)	0.0001
Training						
None	1.73	(0.83, 4.55)	0.45	0.56	(0.18, 1.42)	0.45
Kaizen Basics	1.55	(0.78, 3.86)	0.53	1.19	(0.55, 2.91)	0.83
Leadership	1.52	(0.43, 4.59)	0.72	2.40	(1.01, 7.08)	0.24
Participation						
None (vs. any)	1.23	(0.72, 2.48)	0.82	1.15	(0.47, 0.85)	0.82
Visibility wall	0.75	(0.47, 1.25)	0.83	1.25	(0.73, 2.39)	0.53
5S	0.92	(0.62, 1.34)	0.56	1.28	(0.86, 1.96)	0.69
RPIW	1.06	(0.73, 1.72)	0.68	1.06	(0.72, 1.76)	0.85
Impact of Lean on Workload						
No impact/Decreased	1.0	–	–	1.0	–	–
Increased somewhat	0.68	(0.45, 0.99)	0.24	0.60	(0.38, 0.92)	0.17
Increased substantially	0.55	(0.34, 0.85)	0.08	0.36	(0.20, 0.58)	0.001
First Exposure to Lean						
About 2 years ago	1.0			1.0		
3 or more years ago	1.15	(0.81, 1.63)	0.72	1.42	(0.98, 2.13)	0.32

**Table 7:** Multivariate Multinomial Model: Adjusted Associations between Collective Action (Resources) Item and Demographic, Personal and Practice Variables

	Sufficient resources are available					
	Neutral			Agree		
	Odds Ratio	95% CI	P-value	Odds Ratio	95% CI	P-value
Profession						
Nursing	1.0	–	–	1.0	–	–
Health Care	1.67	(1.15, 2.47)	0.10	1.27	(0.81, 1.97)	0.78
Age group						
≤ 30 years	1.0	–	–	1.0	–	–
31–45 years	1.12	(0.57, 2.24)	0.98	1.13	(0.56, 2.32)	0.98
> 45 years	0.99	(0.44, 2.20)	0.98	0.95	(0.41, 2.25)	0.98
Employment Status						
Full-Time	1.0	–	–	1.0	–	–
Part-Time/Casual	1.03	(0.71, 1.48)	0.98	1.15	(0.75, 1.78)	0.85
Years of experience						
0–5 years	1.0	–	–	1.0	–	–
6–10 years	1.19	(0.61, 2.40)	0.85	0.92	(0.45, 1.84)	0.98
11–15 years	1.29	(0.64, 2.74)	0.76	0.88	(0.40, 1.94)	0.98
15+ years	1.52	(0.77, 3.22)	0.94	1.10	(0.53, 2.43)	0.98
Primary Role						
Direct Care	1.0	–	–	1.0	–	–
Leadership	0.95	(0.49, 1.73)	0.98	0.98	(0.49, 1.83)	0.98
Other	1.52	(0.81, 2.90)	0.67	1.05	(0.46, 2.20)	0.98
Practice Setting						
Acute	1.0	–	–	1.0	–	–
Long-term Care	0.84	(0.49, 1.38)	0.85	1.05	(0.56, 1.83)	0.98
Other	1.00	(0.68, 1.47)	0.98	1.10	(0.70, 1.72)	0.98
Location						
Urban	1.0	–	–	1.0	–	–
Rural/Remote	1.17	(0.84, 1.65)	0.84	1.32	(0.91, 1.94)	0.60
Familiarity with Lean						
Complete	1.0	–	–	1.0	–	–
Somewhat	0.88	(0.60, 1.31)	0.85	0.65	(0.42, 0.98)	0.23
Training						
None (vs. any)	1.33	(0.65, 3.29)	0.85	0.92	(0.33, 3.03)	0.98
Kaizen Basics	1.27	(0.67, 3.11)	0.85	1.52	(0.70, 4.40)	0.78
Leadership	1.95	(0.72, 4.95)	0.60	2.10	(0.94, 5.68)	0.29
Participation						
None (vs. any)	1.35	(0.76, 2.74)	0.28	0.71	(0.27, 1.64)	0.78
Visibility wall	0.92	(0.57, 1.55)	0.85	0.92	(0.55, 1.72)	0.78
5S	0.85	(0.59, 1.29)	0.98	1.26	(0.86, 1.96)	0.98
RPIW	0.65	(0.40, 0.99)	0.78	1.26	(0.81, 1.96)	0.85
Impact of Lean on Workload						
No impact/Decreased	1.0	–	–	1.0	–	–
Increased somewhat	0.62	(0.42, 0.92)	0.14	0.42	(0.26, 0.66)	0.002
Increased substantially	0.46	(0.28, 0.71)	0.01	0.37	(0.21, 0.59)	0.002
First Exposure to Lean						
About 2 years ago	1.0			1.0		
3 or more years ago	0.99	(0.69, 1.42)	0.98	1.22	(0.83, 1.85)	0.81

**Table 8:** Multivariate Multinomial Model: Adjusted Associations between Reflexive Monitoring Item and Demographic, Personal and Practice Variables

	The people I work with believe that Lean is worthwhile					
	Neutral			Agree		
	Odds Ratio	95% CI	P-value	Odds Ratio	95% CI	P-value
Profession						
Nursing	1.0	–	–	1.0	–	–
Health Care	2.60	()1.74, 4.19	0.0002	2.06	(1.21, 3.96)	0.04
Age group						
≤ 30 years	1.0	–	–	1.0	–	–
31–45 years	0.96	(0.46, 1.97)	0.95	2.33	(0.71, 13.2)	0.43
> 45 years	1.06	(0.42, 2.62)	0.95	2.62	(0.69, 17.6)	0.42
Employment Status						
Full-Time	1.0	–	–	1.0	–	–
Part-Time/Casual	0.85	(0.54, 1.30)	0.70	0.81	(0.36, 1.59)	0.80
Years of experience						
0–5 years	1.0	–	–	1.0	–	–
6–10 years	1.12	(0.51, 2.37)	0.87	0.92	(0.31, 3.20)	0.87
11–15 years	0.82	(0.33, 1.91)	0.34	0.72	(0.21, 2.77)	0.95
15+ years	1.84	(0.81, 4.55)	0.95	0.95	(0.32, 3.50)	0.95
Primary Role						
Direct Care	1.0	–	–	1.0	–	–
Leadership	1.70	(0.86, 3.44)	0.31	4.33	(2.24, 9.74)	0.001
Other	0.92	(0.36, 2.05)	0.95	3.74	(1.64, 9.13)	0.01
Practice Setting						
Acute	1.0	–	–	1.0	–	–
Long-term Care	1.51	(0.88, 2.60)	0.34	1.48	(0.57, 3.50)	0.61
Other	1.01	(0.62, 1.62)	0.96	1.04	(0.52, 2.04)	0.95
Location						
Urban	1.0	–	–	1.0	–	–
Rural/Remote	1.81	(1.24, 2.82)	0.01	1.33	(0.75, 2.45)	0.57
Familiarity with Lean						
Complete	1.0	–	–	1.0	–	–
Somewhat	1.20	(0.77, 1.92)	0.70	0.84	(0.45, 1.52)	0.80
Training						
None (vs. any)	2.25	(0.97, 6.63)	0.31	0.29	(0.05, 1.63)	0.31
Kaizen Basics	1.33	(0.62, 4.02)	0.80	0.54	(0.18, 1.81)	0.40
Leadership	3.80	(1.32, 11.6)	0.04	5.21	(2.32, 18.8)	0.004
Participation						
None (vs. any)	1.20	(0.56, 2.41)	0.95	0.75	(0.12, 2.89)	0.44
Visibility wall	0.79	(0.46, 1.40)	0.31	1.07	(0.56, 3.82)	0.95
5S	1.53	(0.93, 2.28)	0.69	1.16	(0.54, 2.17)	0.87
RPIW	0.93	(0.63 1.71)	0.90	1.50	(0.82, 2.84)	0.87
Impact of Lean on Workload						
No impact/Decreased	1.0	–	–	1.0	–	–
ncreased somewhat	0.50	(0.32, 0.75)	0.01	0.47	(0.23, 0.83)	0.04
Increased substantially	0.14	(0.06, 0.24)	0.0001	0.21	(0.08, 0.42)	0.001
First Exposure to Lean						
About 2 years ago	1.0			1.0		
3 or more years ago	0.73	(0.48, 1.09)	0.32	1.06	(0.59, 2.05)	0.95

Professional designation as an HP, in contrast to being a nurse, was also associated with agreement responses to items representing cognitive participation, coherence, reflexive monitoring and collective action (training). HPs were significantly more likely than nurses to support the use of Lean in health care (O.R. =4.11, 95% C.I. 2.70–7.20); to see the potential value of Lean for their work (O.R. =2.57, 95% C.I. 1.73–4.12); to indicate that the people they worked with believe that Lean is worthwhile (O.R. =2.06, 95% C.I. 1.21–3.96); and to believe sufficient training had been provided (O.R. =1.57, 95% CI 1.02–2.45). No differences were identified between HPs and nurses in terms of the sufficiency of resources.

In terms of personal and contextual characteristics, increased workload was strongly associated with weaker normalization across all five models. The associations were particularly negative between somewhat increased workload (O.R. =0.19, 95% C.I. 0.11–0.29) or substantially increased workload and support for the use of Lean (O.R. = 0.05, 95% C.I 0.02–0.09). There were no significant associations between age group, years of professional experience, or employment status and any of the NPT outcome items. Weak but significant associations were noted between several normalization outcomes and: a) practice location (urban vs. rural/remote); and b) employment in long-term care.

Lean Leader training, which focused on Lean tools and methods, was positively associated with all normalization outcomes, except for the provision of sufficient resources. Compared to those without Lean Leader training, respondents with leadership training were more likely to agree that: the people I work with believe Lean is worthwhile (O.R. =5.21, 95% CI = 2.03–13.40); I can see the potential value of Lean for my work (O.R. =3.72, 95% CI = 1.53–18.70); and I support the use of Lean (O.R. = 3.45, 95% CI = 1.24–14.90). Compared to those with any type of training, respondents with no training were much less likely to support the use of Lean in health care (O.*R* = 0.28, 95% CI = 0.10–0.77), but did not differ from those with training in terms of agreement with the other items. Neither completion of Kaizen Basics training nor participation in Lean activities were associated with any of the outcome items.

## Discussion

More than four years after the ambitious and costly launch of Lean as a province-wide strategy to improve health care quality and promote patient-centredness within healthcare in Saskatchewan, the findings of our survey reveal that major gaps remain in embedding the principles and activities of Lean into everyday health care practice, particularly among nurses and direct care providers. Assessing implementation processes of initiatives such as Lean that are ongoing can serve to target issues that require additional support and attention.

Significant differences in responses were evident between: leaders vs. direct care providers; nurses vs. other health professionals; and providers who reported increased workload as a result of Lean vs. those who did not. Respondents in leadership positions were much more likely to view the implementation and benefits of Lean in a positive manner than were direct care providers. More than 75% of respondents indicated that neither sufficient training nor resources (collective action) had been made available to them for the implementation of Lean. Compared to other health care providers, nurses were more likely to report that Lean increased their workload.

The importance of strong leadership in effecting the cultural changes needed for major health reform has been well-recognized [30]. A wide gulf between the perspectives of leaders and direct care providers was apparent, however, with respect to the constructs of coherence, cognitive participation and reflexive monitoring. Based on the premise that senior leaders “are central to ensuring that Lean will pervade the organization’s management system and [to] provide an example of Lean principles to other personnel” [2], major investments had been made by the Ministry of Health to ensure that those in health leadership roles were “committed to learning and applying Lean principles” [2]. These investments at the senior leadership level included a rigorous certification program that included didactic and experiential training, including leadership of Lean events, and an opportunity to visit organizations in the U.S. that have long experience with the use of Lean in manufacturing and health care [31]. Given that only 30% of leaders had Lean Leader training, the basis for the strong support shown by leaders for Lean implementation might be better explained by factors other than training alone. A subsequent paper will compare the narrative responses on the survey between leaders and nurses to shed further light on this finding.

Adequate training and resources are foundational to any attempt to transform health care [32], but the majority of respondents believed these had not been sufficient (Additional file 2: Table S2). Only one quarter of all respondents believed that training had been sufficient and 18% felt that resources had been sufficient to implement Lean. The nature and type of Lean education and participation in Lean activities had interesting implications for the extent of normalization. The majority of respondents reported attending some form of Lean training, but only the variable of having no Lean training at all was associated with (lack of) support for Lean. Kaizen Basics training, which was originally an eight hour introductory program (later scaled back), was intended to provide staff with “a broad overview of Lean principles and methods, so they understand the changes taking place and have a sense of what to expect when they are invited to participate in an improvement event” [33]. This training was attended by three quarters of respondents, but we found no associations between attendance at these sessions and the normalization outcomes examined in this study.

Participation in Lean activities was not associated with the items representing normalization of the Lean approach, in spite of the appeal of providing “hands-on training” as a pedagogical strategy. Our findings contrast with those reported in a recent study examining the introduction of Lean in primary care that noted that the time and intensity of exposure to redesign activities was a positive influence on acceptance by providers [34].

The anticipated benefits of the implementation of Lean are contingent upon widespread dissemination of the philosophy and processes throughout the entire system, resulting in a major cultural shift that focuses on continuous performance improvement [32]. Critical factors such as perceived lack of ownership and subcultural diversity have been noted to derail attempts at transforming culture when direct care providers are not engaged [12]. As recently noted by Bohmer [35], “delivery of care is ultimately governed by structure and process at the ward, clinic or practice level”. Lean implementation in health care can be perceived to compromise professional autonomy [36] and can challenge established professional hierarchy [12], thereby promoting resistance from key stakeholders required to enact the transformation within the system. McIntosh [12] noted that the Saskatchewan government failed to appreciate that key factors such as nurses (and physicians) had the independent ability to push back against this initiative in response to top-down implementation and that Lean “cannot be implemented in the top-down, directive manner displayed to date but must accommodate other powerful decision-makers in these sectors” [12].

Although nurses constitute the largest proportion of licensed health care providers in health care system and their contributions are well-recognized to be pivotal in ensuring patient safety and high-quality care [37], the perspectives of nurse respondents suggest that there are fundamental differences in the way in which Lean impacts the work of nurses compared to other health professionals, despite similarities in Lean training and participation in activities. Nurses play many critical roles in health care delivery, but globally share concerns about understaffing and inadequate training and support [38]. Nurses have been found to encounter an average of 8.4 work system failures in an 8-h shift, a fact compounded by frequent interruptions of their work [39, 40]. Nurses, compared to physicians, have been found to have less work autonomy, fewer professional development opportunities and fewer options for career change [41]; nurses are considered the health professionals most exposed to work strain that compromises their physical and psychological well-being [41, 42]. The implications of Lean for nurses’ work, particularly for bedside nurses, requires further thoughtful consideration. A 2014 survey conducted by the Saskatchewan Union of Nurses [43] reported a statistically significant negative effect of Lean on nurse engagement, usefulness, patient care, time for patient care, workplace issues, availability of supplies, workload, stress and patient care. Our results lend support to the notion of a potential misalignment between the principles and activities of Lean as it had been implemented and the work of clinical nursing, which we are currently investigating in related projects.

Our findings also point to the importance of context in the implementation of Lean. Greater agreement with normalization constructs was noted for non-urban respondents, whose practice involves different demands than larger urban settings and highlights again the importance of context in introducing system-wide change. Context is a critical consideration in the implementation of large-scale interventions [44–47], with “dynamic elements of context play[ing] a powerful role in shaping participants’ capacity and potential” [46] Complex interventions that cannot be integrated smoothly within organizational contexts and do not prove to be workable alongside other tasks and duties are unlikely to be normalized [48]. Conversely, adaptations to organizational contexts and tasks and duties may act to facilitate the normalization of interventions such as Lean.

Our results highlight the importance of attending to increased job demands that may result from new interventions. Job demands are aspects of work associated with physical, emotional and cognitive efforts and can translate into job stressors and burn-out if sufficient resources are not available [48]. Perceived increased workload was the only variable to be a significant predictor of negative responses across all five of the models. The implementation of Lean was reported to have different implications for the workload of individual respondents, pointing to the need for leaders to carefully evaluate how interventions may impact upon the workload of their subordinates.

The use of Lean as a large-scale quality improvement strategy has generated controversy on multiple fronts, but evidence on outcomes of Lean implementation is still emerging. Batalden et al. [49] have questioned the wisdom and appropriateness of implementing quality improvement systems such as Lean which are underpinned by “goods-dominated” logic systems in health care. Improvement strategies that recognize the dynamic environment of health care provision, where goods and services are consumed and produced simultaneously, may be more easily integrated into health care practice.

### Limitations

While our cross-sectional survey findings include the responses of over 1000 health care professionals with a wide range of professional roles, diverse practice settings and geographic locations, we acknowledge the potential for non-response bias resulting from the lower than desired response rates. Because reasons for nonresponse are not known to researchers, one strategy to assess the effect of non-response bias is to analyze the known demographic or organizational characteristics of the population [50]. The demographic profile of respondents in this survey reflects some of the key aspects of the Canadian healthcare workforce. Eighty per cent of all health care providers in Canada are female with an average age of 43 years [29], although the proportion of nurses was over-represented in this survey, given that registered nurses, licensed practical nurses and registered psychiatric nurses account for slightly more than one-third of all Canadian health care workers [29]. Within the province of Saskatchewan, the 2011 census indicates that one-third of the population resides in a rural location [51], suggesting that the views of rural health care professionals (41.4%) may have also been over-represented.

Sampling bias was mitigated by having professional associations distribute the invitation to participate to all members, with the exception of Registered Nurses whose members could choose to opt out of surveys. According to Dillman and colleagues [52], our sample sizes of 734 nurses out of 10,000 possible respondents and 298 HPs out of 1219 possible respondents (excluding physicians) achieved the completed sample size necessary for a ± 5% margin of error in both groups. Strategies to maximize response rates, such as having the associations send reminders [49], were employed.

The decision to recruit professionals through their licensing bodies for this online survey was made consciously to maintain independence of the survey from those with vested interests in the outcomes of evaluation, such as employers, government or unions. Because exposure to Lean had been widespread throughout Saskatchewan, it was not possible to identify a priori individuals who were actually engaged in Lean implementation versus those who were not in order to better tailor the sampling frame. Potential explanations for the low response rate are myriad - mode of administration, time of year, survey fatigue, email or work overload, and perceived lack of relevance are just a few possibilities. Surveys, as a research strategy, are recognized to be challenged by declining response rates throughout developed countries [53]. In spite of these issues, the survey method allowed us to hear the voices of a broad cross-section of health providers across the province in a manner other research designs would not have.

Over 300 respondents chose not to identify their profession, but went on to complete the remainder of the survey. Their responses were not included in this analysis, but subsequent analysis is planned to compare responses between those who did and did not list their professions. Although Lean reforms were putatively meant as a vehicle to empower both patients and workers [12], the highly polarized nature of the debate surrounding Lean in health care, an atmosphere of mistrust in health care and fear of reprisal [54] from employers may have contributed to the reluctance to identify profession on the survey. Hearing the voices and perspectives of health care providers is essential to authentic and sustainable transformation of health care [55]. Creating opportunities to engage direct providers in reform would support achievement of this large-scale transformation.

Low rates of participation on the part of physicians and subsequent exclusion of their perspectives on this topic also limits the conclusions we can draw from this cross-sectional survey. Physicians’ lack of participation was unfortunate, but not surprising, in a professional group recognized to have low survey response rates [56] and when the regulations of their professional association did not allow for the individual email contacts that were sent to the other groups of health care providers. Physician leadership and engagement has been noted as a strong lever for driving interdisciplinary work forward [57] and are critical to the successful implementation of health care reforms [58], making their viewpoints central to assessment of implementation of complex interventions such as Lean. Further work is needed to identify strategies to hear the perspectives of physicians.

The outcomes for this study were selected to reflect the extent to which Lean had been embedded in Saskatchewan health care. While a number of survey instruments were evaluated for use in this study, the NoMAD was selected on the basis of its strong conceptual underpinnings and relevance to our objectives. Currently undergoing psychometric assessment (T. Finch, personal communication, September 2016), the NoMAD requires additional evaluation to guide overall and subscale scoring and interpretation. Because each of the items was considered to have face validity and reflected a key aspect of normalization important for assessment in this study and because scoring directions for the NoMAD are not yet available, the decision was made to conduct the analyses using individual items.

## Conclusions

Normalization Process Theory offered a feasible and highly applicable model through which to assess implementation processes of the province-wide adoption of Lean. Use of a modified NoMAD survey allowed us to capture key elements of implementation that were of primary interest. The findings of this project provide a window into the perspectives of health care professionals who are in the midst of undergoing large scale transformation of a health care system and can inform future research, practice and policy related to implementation processes.

The implementation of Lean in Saskatchewan health care represents an ambitious, high-investment, multi-pronged attempt at a large-scale transformation “aimed at coordinated, system-wide change affecting multiple organizations and care providers” [4]. Recognizing that successful transformation is the result of constant small-scale changes to structures and process over long periods of time, Bohmer [35] suggests that the actual model of quality improvement (Lean or alternatives) is less important to achieving the goals of health reform than internalized repetitive and consistent process. The findings of our survey highlight that, while substantial progress has been made in the province-wide effort to implement, embed and integrate Lean into the Saskatchewan health care system over the past few years, ongoing support and innovative implementation strategies will be required if large-scale quality improvement initiatives are to become a routine part of practice for health care providers across the province and produce the intended improvements in quality and patient-centredness.

## Additional files

Additional file 1: **Table S1.** Normalization Process Theory Core Construct. (DOCX 13 kb)

Additional file 2: **Table S2.** Responses by Professional Group. (DOCX 12 kb)

Additional file 3: **Table S3.** Revised NoMAD Survey Responses by Profession. (DOCX 14 kb)

## Abbreviations

CI: Confidence Interval
NPT: Normalization Process Theory
OR: Odds Ratio
US: United States
