# Supplementary material for: Assessing the implementation processes of a large-scale, multi-year quality improvement initiative: survey of health care providers
Source: BMC Health Serv Res. 2018 Apr 3;18:237. doi: 10.1186/s12913-018-3045-6 (PMC5883256; doi:10.1186/s12913-018-3045-6)
Supplement: Supplementary file 1 — Table S1. Normalization Process Theory Core Construct. (DOCX 13 kb) [file 12913_2018_3045_MOESM1_ESM.docx]

Supplementary Table 1. Normalization Process Theory Core Constructs^15^

| Core Construct | Definition | Components |
| --- | --- | --- |
| Coherence | Sense-making work done individually and collectively during operationalization of a set of practices | - Differentiation - Communal specification - Individual specification - Internalization |
| Cognitive Participation | Relational work done to build and sustain a community of practice around a new practice | - Initiation - Enrolment - Legitimation - Activation |
| Collective Action | Operational work done to enact of new set of practices | - Interactional workability - Relational integration - Skill set workability - Contextual integration |
| Reflexive Monitoring | Appraisal work done to assess and understand the effects of new practices on themselves and others around them | - Systematization - Communal appraisal - Individual appraisal - Reconfiguration |
