# Supplementary material for: Assessing the implementation processes of a large-scale, multi-year quality improvement initiative: survey of health care providers
Source: BMC Health Serv Res. 2018 Apr 3;18:237. doi: 10.1186/s12913-018-3045-6 (PMC5883256; doi:10.1186/s12913-018-3045-6)
Supplement: Supplementary file 2 — Table S2. Responses by Professional Group. (DOCX 12 kb) [file 12913_2018_3045_MOESM2_ESM.docx]

Supplementary Table 2. Responses by Professional Group

| Professional Group | N | Responses Received | Responses Included |
| --- | --- | --- | --- |
| Nurses | | | |
| Registered Nurses | 4,800 | 396 (8.3%) | 395 (8.3%) |
| Licensed Practical Nurses | 3,400 | 315 (9.3%) | 314 (9.3%) |
| Registered Psychiatric Nurses | 879 | 25 (2.8%) | 25 (2.8%) |
| Health Professionals | | | |
| Physiotherapists | 600 | 67 (11.2%) | 67 (11.2%) |
| Occupational Therapists | 210 | 56 (26.7%) | 56 (26.7%) |
| Physicians | 2,144 | 36 (1.7%) | Excluded |
| Hospital-Based Pharmacists | 264 | 131 (49.6%) | 131 (49.6%) |
| Dietitians | 355 | 44 (12.4%) | 44 (12.4%) |
| Unspecified Profession | | | |
| Unspecified |  | 308 | 0 |
