# Supplementary material for: Assessing the implementation processes of a large-scale, multi-year quality improvement initiative: survey of health care providers
Source: BMC Health Serv Res. 2018 Apr 3;18:237. doi: 10.1186/s12913-018-3045-6 (PMC5883256; doi:10.1186/s12913-018-3045-6)
Supplement: Supplementary file 3 — Table S3. Revised NoMAD Survey Responses by Profession. (DOCX 14 kb) [file 12913_2018_3045_MOESM3_ESM.docx]

Supplementary Table 3. Revised NoMAD Survey Responses by Profession

|  | Nursing  N= 734 (%) | | | Health Professionals  N=298 (%) | | | P-value |
| --- | --- | --- | --- | --- | --- | --- | --- |
|  | Agree | Neutral | Disagree | Agree | Neutral | Disagree |  |
| Coherence | | | | | | | |
| 1. I can see how Lean differs from usual ways of working | 361 (49.2) | 194 (26.4) | 179 (24.4) | 165 (55.4) | 75 (25.2) | 58 (19.5) | 0.14 |
| 2. The people I work with have a shared understanding of the purpose of Lean | 265 (36.2) | 171 (23.3) | 297 (40.5) | 121 (40.6) | 85 (28.5) | 92 (30.9) | 0.01 |
| 3. I understand how Lean affects the nature of my own work | 385 (52.5) | 173 (23.6) | 175 (23.9) | 160 (53.7) | 87 (29.2) | 51 (17.1) | 0.03 |
| 4. I can see the potential value of Lean for my work | 180 (24.6) | 137 (18.7) | 416 (56.8) | 138 (46.3) | 80 (26.9) | 80 (26.9) | 0.0001* |
| Cognitive Participation | | | | | | | |
| 1. There are key people who drive Lean forward and get others involved | 428 (58.3) | 138 (18.8) | 168 (22.9) | 202 (67.8) | 70 (23.5) | 26 (8.7) | 0.0001* |
| 2. One of my professional roles is participating in Lean activities | 326 (44.4) | 179 (24.4) | 229 (31.2) | 116 (38.9) | 98 (32.9) | 84 (28.2) | 0.02 |
| 3. I am open to working with colleagues in new ways to use Lean | 312 (42.5) | 234 (31.9) | 188 (25.6) | 183 (61.4) | 83 (27.9) | 32 (10.7) | 0.0001* |
| 4. I support the use of Lean in health care | 131 (17.9) | 150 (20.4) | 453 (61.7) | 121 (40.6) | 90 (30.2) | 87 (29.2) | 0.0001* |
| Collective Action | | | | | | | |
| 1. I can easily integrate Lean into my existing work | 128 (17.4) | 206 (28.1) | 400 (54.5) | 89 (29.9) | 116 (38.9) | 93 (31.2) | 0.0001* |
| 2. Lean disrupts my relationships with the people I work with | 365 (49.7) | 220 (30.0) | 149 (20.3) | 81 (27.2) | 104 (34.9) | 113 (37.9) | 0.0001* |
| 3. I am confident in the skills of people leading the use of Lean | 113 (15.4) | 181 (24.7) | 440 (60.0) | 70 (23.5) | 98 (32.9) | 130 (43.6) | 0.0001* |
| 4. Sufficient training is provided to enable health care providers to implement Lean in health care | 154 (21.0) | 184 (25.1) | 396 (54.0) | 78 (26.2) | 114 (38.3) | 106 (35.6) | 0.0001* |
| 5. Sufficient resources are available to support the implementation of Lean in health care | 122 (16.6) | 160 (21.8) | 451 (61.5) | 61 (20.5) | 103 (34.6) | 134 (45.0) | 0.0001* |
| 6.Management adequately supports the use of Lean in health care | 306 (41.8) | 229 (31.2) | 198 (27.0) | 131 (44.0) | 111 (37.3) | 56 (18.8) | 0.02 |
| Reflexive Monitoring | | | | | | | |
| 1. I am aware of reports about the outcomes of Lean implementation in Saskatchewan | 478 (65.1) | 129 (17.6) | 127 (17.3) | 194 (65.1) | 61 (20.5) | 43 (14.4) | 0.36 |
| 2. I am aware of reports about the outcomes of Lean implementation outside of the province | 253 (34.5) | 192 (26.2) | 289 (39.4) | 95 (31.9) | 74 (24.8) | 129 (43.3) | 0.50 |
| 3. The people I work with believe that Lean is worthwhile | 58 (7.9) | 103 (14.0) | 573 (78.1) | 41 (13.8) | 97 (32.6) | 160 (53.7) | 0.0001* |
| 4. I value the effects that Lean has had on my work | 96 (13.1) | 151 (20.6) | 487 (66.4) | 77 (25.8) | 114 (38.3) | 107 (35.9) | 0.0001* |
| 5. Feedback about Lean can be used to improve its implementation in health care in the future | 316 (43.1) | 174 (23.7) | 244 (33.2) | 187 (62.8) | 73 (24.5) | 38 (12.8) | 0.0001* |
| 6. I can modify how I use Lean in my work | 190 (25.9) | 249 (33.9) | 295 (40.2) | 138 (46.3) | 114 (38.3) | 46 (15.4) | 0.0001* |
